# Supplementary material for: Reactive Extrusion and Magnesium (II) N-Heterocyclic Carbene Catalyst in Continuous PLA Production
Source: Polymers (Basel). 2019 Dec 2;11(12):1987. doi: 10.3390/polym11121987 (PMC6960517; doi:10.3390/polym11121987)
Supplement: Supplementary file 1 [file polymers-11-01987-s001.pdf]

Reactive extrusion and magnesium (II) N-heterocyclic carbene catalyst in continuous PLA production

Rosica Mincheva, Satya Narayana Murthy Chilla, Richard Todd, Brieuc Guillerm, Julien De Winter, Pascal Gerbaux, Olivier Coulembier, Philippe Dubois, Jean-Marie Raquez*

Correspondence to: Jean-Marie Raquez (E-mail: jean-marie.raquez@umons.ac.be) Laboratory of Polymeric and Composite Materials, University of Mons, 23 Place du Parc, B-7000, Mons, Belgium

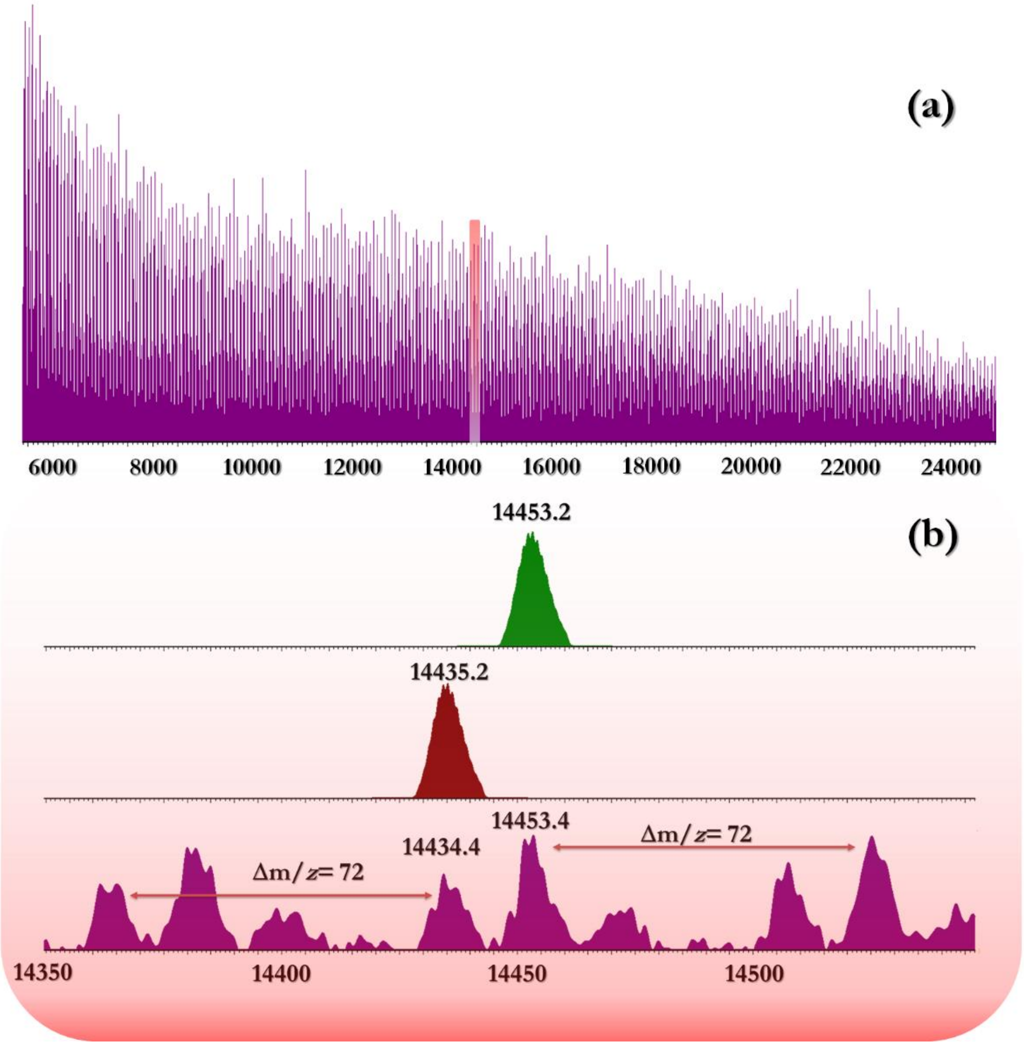

FIGURE S1 (a) global MALDI mass spectrum recorded for poly(L-lactide) (Table 2, Entry 3) and (b) magnification between m/z 14350 and m/z 14550 with theoretical isotopic model for cyclic (red) and linear (green) (HO- and -COOH as end-groups) polymer with 100 lactoyl monomer units respectively.

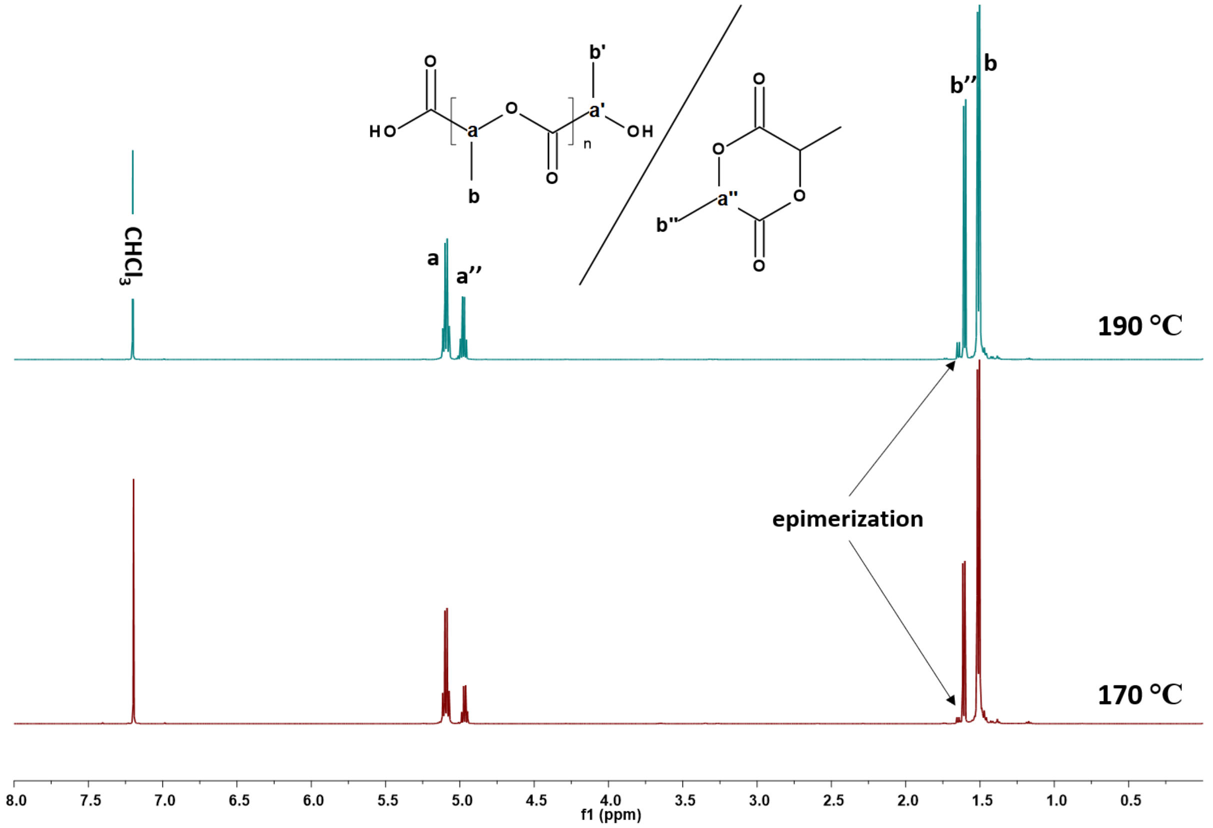

FIGURE S2 ^{1}H NMR of PLA as obtained after 30 min at 170 (red curve) or 190 °C (bleu curve) in batch scale (Table 2, Entry 3 and 8).

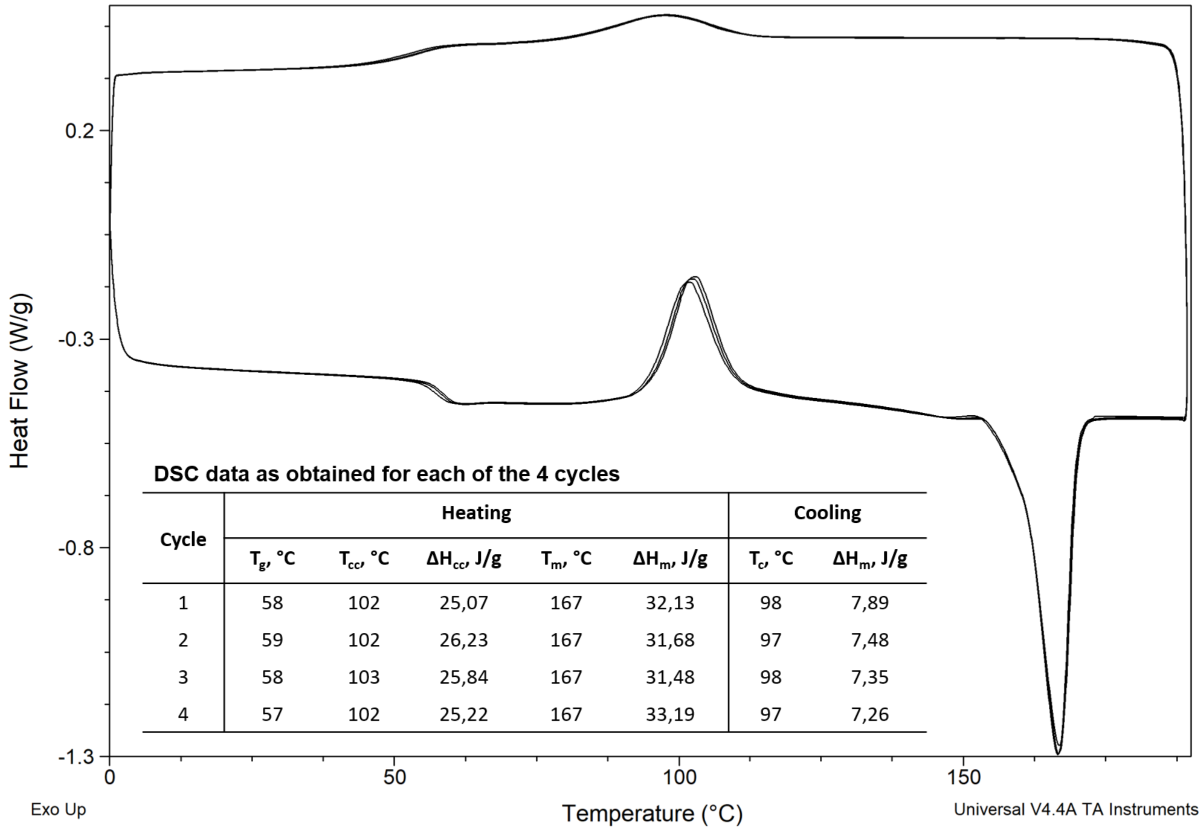

DSC data as obtained for each of the 4 cycles

Cycle	Heating					Cooling	
	T_g, °C	T_{cc}, °C	ΔH_{cc}, J/g	T_m, °C	ΔH_m, J/g	T_c, °C	ΔH_m, J/g
1	58	102	25,07	167	32,13	98	7,89
2	59	102	26,23	167	31,68	97	7,48
3	58	103	25,84	167	31,48	98	7,35
4	57	102	25,22	167	33,19	97	7,26

FIGURE S3 Four-cycle DSC analyses of PLA obtained after 30 min at 170 °C in batch scale (Table 2, Entry 3) after monomer purification.
